# Studying the integrated functional cognitive basis of sustained attention with a Primed Subjective-Illusory-Contour Attention Task

**DOI:** 10.1038/s41598-018-31876-7

**Published:** 2018-09-10

**Authors:** Benjamin Ultan Cowley

**Affiliations:** 1 Cognitive Science, Department of Digital Humanities, Faculty of Arts, University of Helsinki, Helsinki, Finland; 2 Cognitive Brain Research Unit, Department of Psychology and Logopedics, Faculty of Medicine, University of Helsinki, Helsinki, Finland

## Abstract

Sustained attention plays an important role in everyday life, for work, learning, or when affected by attention disorders. Studies of the neural correlates of attention commonly treat sustained attention as an isolated construct, measured with computerized continuous performance tests. However, in any ecological context, sustained attention interacts with other executive functions and depends on lower level perceptual processing. Such interactions occur, for example, in inhibition of interference, and processing of complex hierarchical stimuli; both of which are important for successful ecological attention. Motivated by the need for more studies on neural correlates of higher cognition, I present an experiment to investigate these interactions of attention in 17 healthy participants measured with high-resolution electroencephalography. Participants perform a novel 2-alternative forced-choice computerised performance test, the Primed Subjective Illusory Contour Attention Task (PSICAT), which presents gestalt-stimuli targets with distractor primes to induce interference inhibition during complex-percept processing. Using behavioural and brain-imaging analyses, I demonstrate the novel result that task-irrelevant incongruency can evoke stronger behavioural and neural responses than the task-relevant stimulus condition; a potentially important finding in attention disorder research. PSICAT is available as an open-source code repository at the following url, allowing researchers to reuse and adapt it to their requirements. https://github.com/zenBen/Kanizsa_Prime/.

## Introduction

Sustaining attention on task is an important skill for functioning in, e.g. education or the workplace. Clinical pathologies of attention are recognised as a growing problem in children and adults^1^ (for US Centres of Disease Control demographic statistics, see^2^); thus, better understanding and measurement of sustained attention is important for basic research and applications. Sustained attention, also termed vigilant attention, is continuous awareness of a subset of possible stimuli, that is self-directed, task-relevant, and occurs when responding to approximately uniform task stimulation^3^. For example, reading a textbook tends to require sustained attention, whereas reading a flashing advertisement sign is governed by other forms of attention. Given the interaction of bottom-up and top-down influences on attention, it is important to consider to what is attention being paid. Measurement and testing is often done with a computerised continuous performance test (CPT), such as the Test Of Variables of Attention (T.O.V.A.). Such ‘gold standard’ CPTs attempt to index sustained attention by isolating it as a top-down mechanism, using a simple repetitve classification task that strictly controls for cognitive/perceptual complexity by simplifying the stimulus presentation (for example, T.O.V.A. uses a monochrome rectangular stimuli, white on black, altering only the vertical offset of the white rectangle for the classification task).

However, while such strictly-controlled CPTs may test sustained attention as an isolated construct, it is not isolated in any ecological context. Sustained attention integrates with other executive functions and depends on lower level perceptual processing^4^. Here, I report a study of two important demands of ecological attention: (a) successful attending may require inhibition of interference; (b) natural target stimuli are complex and often noisy.

To examine these interactions of attention, I conducted an experiment to probe ecological sustained attention and its neural correlates by measuring healthy participants with high-resolution electroencephalography (EEG) while they perform a novel CPT. This CPT uses gestalt-image targets, primed by congruent or incongruent distractors, to induce ecological sustained attention requiring demands (a) and (b) above. It is thus called the Primed Subjective Illusory Contour Attention Task (PSICAT).

The primary research question of this experiment is whether task-irrelevant (in)congruency – in the form of an intra-modal distractor prime – can evoke stronger behavioural and neural responses than the task-relevant stimulus condition. Primers are attention-grabbing but irrelevant to the task, and in a strategic sense should be ignored, because only target-recognition determines performance. However the primers are displayed in the same modality as the targets, making them difficult to ignore, and thus their impact will be much larger than their utility to the participant requires. It is thus of basic research interest to compare observations of task-relevant gestalt-target conditions to task-irrelevant congruency conditions. I predicted that congruency effects will be much larger than gestalt-value effects due to their greater adaptive value.

## Protocol

I briefly describe the novel task to elucidate the experiment. In order to provide ‘approximately uniform task stimulation’^3^, a test of sustained attention should not contain extensive temporal structure; in other words, should not evolve any narrative from beginning to end. Respecting this, the PSICAT protocol retains the standard repetitve classification-task structure, and adds complex target stimuli preceded by either congruent or incongruent interference primers. PSICAT thus aims to probe the neural correlates of sustained attention by employing stimuli, primed Kanizsa shapes, which require integrative cognitive processing.

As targets, PSICAT uses the Kanizsa subjective contour illusion (SCI; see *Methods* for an illustrative figure), which is a perceived polygon induced by collinear ‘Pac-Man’ shapes at the vertices. When the Pac-Man shapes are not collinear, the stimulus forms a no-SCI target. PSICAT is a two-alternative forced-choice task, where the response is a left or right hand button-press to the presence or absence of an SCI in Kanizsa stimuli. The classification required by the task involves discrimination of gestalt images from images with identical visual features but no gestalt property. Congruency of primers creates a task-irrelevant probe condition. Thus, PSICAT protocol consists of 2 × 2 conditions (of primary interest): congruent SCI, incongruent SCI, congruent no-SCI, incongruent no-SCI (see *Methods* for an illustrative figure).

## Research Questions

To address the main research question, I provide evidence by comparing relative effect sizes (ES) for tests on the task-relevant stimulus conditions (SCI vs no-SCI) against ES for tests on task-irrelevant (in)congruency conditions. These tests compare the effect of conditions (independent variable) on behavioural and brain-imaging data (dependent variables) recorded during performance of PSICAT. These tests describe the effects of the task conditions, and final analysis is then a direct comparison of the standardised magnitudes of those effects.

### Behaviour

Comparing to incongruent trials, I predicted congruent trials to show faster response times (RTs) and reduced error rates, because they require no inhibition of initial neural response signals, and the priming gives an accuracy advantage. Compared to no-SCIs, the SCI targets should produce faster RTs (shown by Tanskanen *et al*.^5^), and reduced response time variability (RTV) (shown by Marini and Marzi^6^).

### Neural Correlates

Both oscillations^7,8^ and event-related potentials (ERPs)^9,10^ of the EEG have been related to attention processes.

Marini and Marzi^6^ showed that early ERP components N1, N2pc, P3a respond to SCIs with larger amplitude than for control stimuli. Following this, I predicted that N1 (from primers and targets) and P3a (from targets only, because P3a from primers would overlap targets) should be larger following SCIs (than no-SCIs). And I predicted a large effect due to expectation contradiction: that after incongruent trials, the ‘novelty’ P3b should be larger than similar-target congruent trials^10^.

Regarding oscillations, based on^7,8^ I predicted that sustained attention would affect the EEG as an increase in fronto-medial (FM) theta-band power *at the end of the test*, compared to the beginning^7,8^.

## Results

17 neurologically-healthy, unmedicated, right-handed participants (11 females, six males) were recorded with 128-channel EEG during ~22 mins of task performance (see *Methods* for complete details).

In order to aid presentation and discussion, I give each research question/test a name and formal hypothesis. I also state each as an inequality using abbreviated variable names. The same abbreviations are used in figures and tables to report results. In addition to abbreviations already defined, these include: *con* congruent; *inc* incongruent; *pri* primer stimuli; *tgt* target stimuli. Thus, the main research question is stated formally as:

**HES** Standardised ES of congruent vs incongruent tests will be larger than standardised ES of SCI vs no-SCI tests; thus *ES*.*conVinc* > *ES*.*SCInoSCI*.

The subtest names/hypotheses are given below. Across the results I have referred to statistical significance levels by the common coding: *p* ≤ 0.1, *p ≤ 0.05, **p ≤ 0.01, ***p ≤ 0.001, ****p ≤ 0.0001.

### Behavioural Results

Following the behaviour predictions above, I define two hypotheses related to response time (H1), and one for error rate (H2):

**H1rt** group mean RTs will be shorter for congruent vs incongruent conditions; and for SCI vs no-SCI conditions moderated by congruency; therefore *con*.*SCI*.*rt* < *con*.*noSCI*.*rt* < *inc*.*SCI*.*rt* < *inc*.*noSCI*.*rt*.

**H1rtv** group mean RTV will be lower for SCI vs no-SCI conditions; therefore *SCI*.*rtv* < *noSCI*.*rtv*.

**H2err** error rate will be lower comparing congruent to incongruent conditions; therefore *con*.*error* < *inc*.*error*.

Median RTs, RTVs, and error rates per condition were: *con*.*SCI* (396 ms, 65 ms, 0.7%); *con*.*noSCI* (453, 101, 0.7); *inc*.*SCI* (468, 78, 2); *inc*.*noSCI* (489, 85, 0.8).

**Figure 1:**
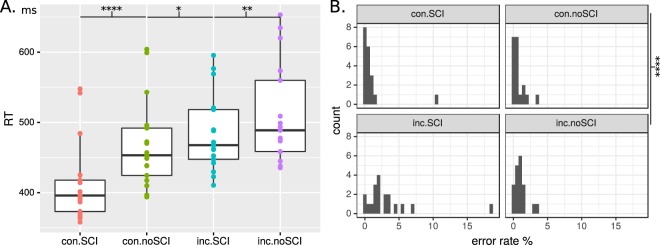
Panel (A) RTs in ms for hit trials in each primary condition (L to R: congruent SCI, congruent no-SCI, incongruent SCI, incongruent no-SCI); points are median RTs per participant; boxes show: sample median, first and third quartiles, and whiskers at 1.5× inter-quartile range. There is a clear condition-wise trend: RTs increase first by congruency and then by SCI. Panel (B) histograms of error rates per condition. Error rates are uniformly low, excepting inc.SCI condition where several participants had around 5% error; and one individual’s responses to SCI targets had (relatively) high error.

Condition RTs were ordered as expected *con*.*SCI*.*rt* < *con*.*noSCI*.*rt* < *inc*.*SCI*.*rt* < *inc*.*noSCI*.*rt*, as shown in Fig. 1, Panel A. Note that these relationships are *transitive*). Thus condition *con*.*SCI* not only has shorter RTs than the condition it is tested against, *con*.*noSCI*, but each other condition too. This implies that the comparison of pooled conditions can be inferred, i.e. congruent RTs are (statistically significantly) less than incongruent, and *inc*.*noSCI* RTs are greater than all other conditions combined. The latter implies that the effect of an SCI primer magnifies the effect of incongruency, in addition to obviously enlarging the speed of recognition of an SCI target.

Further, condition of target SCI versus no-SCI followed the predicted inequality for RTV, *SCI*.*rtv* < *noSCI*.*rtv*; as did condition of congruent versus incongruent trials for errors, *con*.*err* < *inc*.*err* (see Fig. 1, Panel B). All condition differences of behaviour were significant (after multiplicity adjustment) for each predicted inequality as shown in Table 1, indicating support for **H1rt**, **H1rtv**, and **H2err**.

**Table 1:** Hypothesis test results for **H1rt**, **H1rtv**, and **H2err**.

Hyp.	Condition comparison	Med. diff	*U*	adj. *p*	ES
**H1rt**	*con*.*SCI*.*rt* < *con*.*noSCI*.*rt*	51 ms	0	4 × 10^−5^	0.99
**H1rt**	*con*.*noSCI*.*rt* < *inc*.*SCI*.*rt*	19 ms	38	0.04	0.51
**H1rt**	*inc*.*SCI*.*rt* < *inc*.*noSCI*.*rt*	22 ms	20	0.008	0.64
**H1rtv**	*SCI*.*rtv* < *noSCI*.*rtv*	14 ms	161	0.02	0.57
**H2err**	*con*.*err* < *inc*.*err*	1.1%	1	6 × 10^−5^	0.97

Columns are: **Hyp**. Hypothesis; **Condition comparison**; **Med**. **diff** median difference of conditions; **U** Mann-Whitney U-test statistic; **adj**. **p** Bonferroni-Holm adjusted *p*-values; **ES** effect size.

### Neural Results

#### ERP Results

Based on the predictions above, I use early ERP components to probe participants’ processing of congruency (H3) and SCIs (H4), with the following hypotheses:

**H3P3a** P3a will have more positive amplitude for congruent vs incongruent conditions; therefore *con*.*P*3*a* > *inc*.*P*3*a*.

**H3P3b** P3b will have more positive amplitude for incongruent vs congruent conditions; therefore *con*.*P*3*b* < *inc*.*P*3*b*.

**H4PN1** In response to primers, visual N1 will have more negative amplitude in SCI than in nonSCI trials; therefore *pri*.*SCI*.*N*1 < *pri*.*noSCI*.*N*1.

**H4tN1** In response to targets (corrected for primer offsets), visual N1 will have more negative amplitude in SCI than in nonSCI trials; therefore (in each congruency condition): *tgt*.*SCI*.*N*1 < *tgt*.*noSCI*.*N*1.

**H4tP3** In response to targets (corrected for primer offsets), P3a will have more positive amplitude in SCI than in nonSCI trials; therefore (in each congruency condition): *tgt*.*SCI*.*P*3*a* > *tgt*.*noSCI*.*P*3*a*.

Note that because trials have two parts (primer and target), there are two separate *classes* of ERPs: for the whole trial, or for the two parts separately; depending on the hypothesis. Specifically, questions of congruency (H3) involve both primer and target; ERPs for congruency treat a whole trial as a single event and use a baseline from the pre-primer period. Questions of gestalt shape (H4) require treating neural responses to primers and targets as separate events. Thus the baseline for target responses (H4t) come from the period immediately before the target, to account for effect of primers. See Methods for exact details.

**Figure 2:**
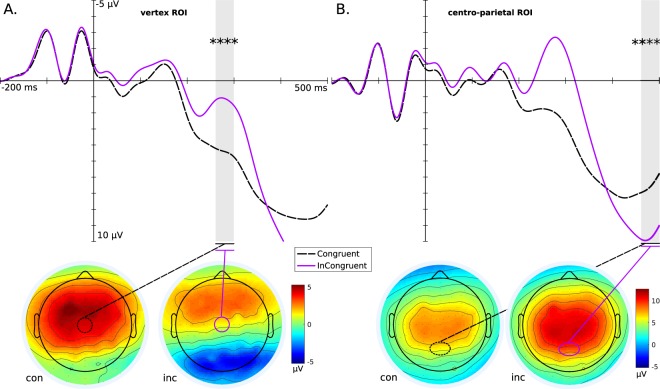
Spatial and temporal activations in congruent and incongruent conditions shown by P300 grand average ERPs (calculated for ROIs) and associated whole-head scalp maps (calculated for the time window of curve testing) to show the spatial extent of the ROI. Significance levels: ****p ≤ 0.0001 Panel (A) P3a at the vertex ROI, significantly larger amplitude for congruent than incongruent within the grey highlighted time-window, with condition-wise scalp maps. Panel (B) P3b at the centro-parietal ROI, significantly larger amplitude for incongruent than congruent within the grey highlight, with condition-wise scalp maps.

Table 2 shows the results of ERP tests for hypothesis sets 3 and 4. The significance testing shows strong support for **H3P3a** and **H3P3b**. The P3a recorded at the vertex ROI, and P3b at centro-parietal ROI, are shown in Fig. 2 (panel A and B, respectively). Both panels also show the associated scalp maps for the time window of testing, indicating the spatial activation during P3a,b and the context of the ROIs.

**Table 2:** Significance tests for the condition-wise difference of mean amplitude ($\bar{\mu }V$) within time windows corresponding to ERP components.

Hyp.	Cnd.	ERP	ROI	Diff $\bar{\mu }V$	*t*	df	adj. *p*	ES
**H3P3a**	—	P3a	vertex	3.23	6.68	33	5 × 10^−7^	2.29
**H3P3b**	—	P3b	parietal	−3.09	−5.67	33	8 × 10^−6^	−1.94
**H4pN1**	*pri*	N1	occipital	−1.30	−4.49	33	2 × 10^−4^	−1.54
**H4tN1**	*con*	N1	occipital	1.67	3.54	16	0.99	—
**H4tN1**	*inc*	N1	occipital	−1.59	−3.06	16	0.01	−1.05
**H4tP3a**	*con*	P3a	vertex	1.63	2.29	16	0.05	0.79
**H4tP3a**	*inc*	P3a	vertex	1.00	1.76	16	0.09	0.6

Columns are: **Hyp**. hypothesis; **Cnd**. congruency condition (only for tests of SCI vs no-SCI); **ERP** tested component; **ROI** centre of region of interest; **Diff** $\bar{\mu }V$ condition-wise difference of mean amplitude in microvolts; **t** t-statistic; **df** degrees of freedom; **adj**. **p** Bonferroni-Holm adjusted *p*-value; **ES** Cohen’s *d* effect size.

**Figure 3:**
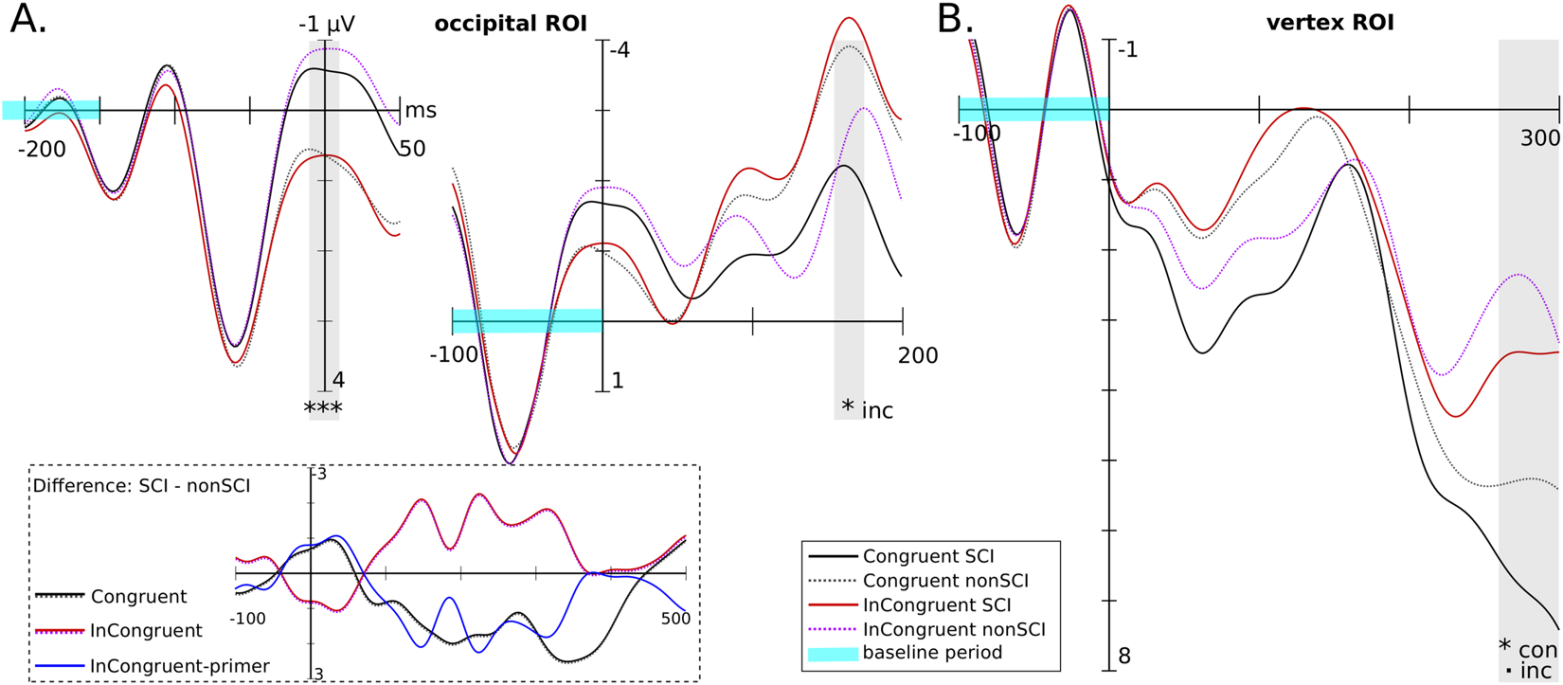
Early components in response to gestalt/non-gestalt stimulus conditions shown by grand average ERPs calculated over ROIs. Significance levels: *p* ≤ 0.1, *p ≤ 0.05, ***p ≤ 0.001 Panel (A) N1 at the occipital ROI for primer (left) and target (right) (note each plot uses different baseline period). To clarify the relation between conditions, the inset box shows the difference wave across the whole target period. N1 has significantly larger amplitude for SCI than non-SCI within the grey highlighted time-windows, in response to primers and congruent targets, *but the pattern is reversed for incongruent targets*. Panel (B) P3a at the vertex ROI, showing significantly larger amplitude for SCI than non-SCI within the grey highlight (marginal for incongruent).

The tests also show partial support for the relatively greater effect of SCIs on early components. **H4pN1** is strongly supported so primer-condition is definitely relevant to the processing of subsequent targets. **H4tN1** is supported but only for the incongruent condition. The congruent condition difference is similarly large but has opposite sign to what was expected, thus non-significant under one-tailed test. The test for **H4tP3a** was significant for congruent condition, and marginal for incongruent, but with only small effect size. Early components are illustrated in Fig. 3 below.

#### Oscillatory Results

The effect of sustained attention on oscillatory EEG^7,8^ is examined via spectral analysis and event-related spectral perturbations (ERSPs), to address the hypothesis:

**H5fmt** FM theta-band power will be greater in the last 10% of trials compared to the first 10% of trials; thus *fmθ*.*first*10*pc* < *fmθ*.*last*10*pc*.

**Figure 4:**
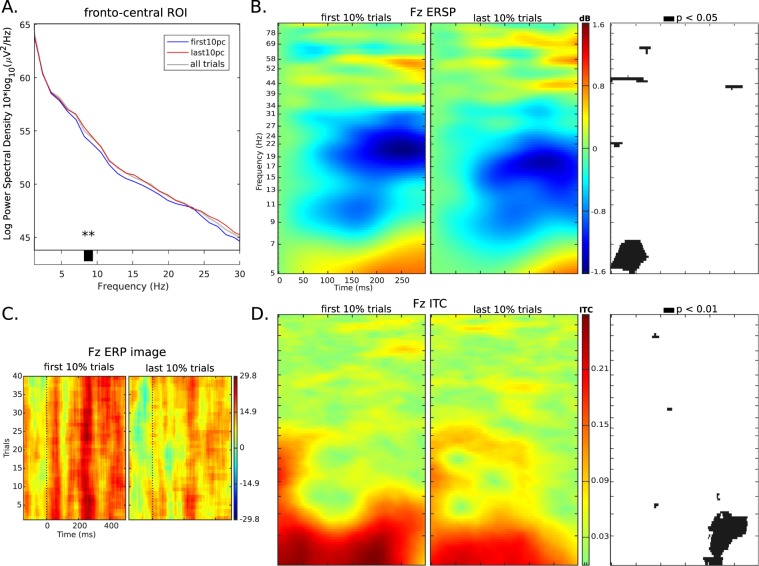
Spectral power comparisons between first 10% and last 10% of trials, at fronto-medial electrodes. Panel (A) The FFT power spectrum from 1–30 Hz shows that FM theta band power is significantly higher in the late (red line) than early (blue line) subset of trials (by permutation testing). The spectrum of all trials (grey line) is included for comparison, and lies mostly between the other spectra. Panel (B) The ERSP indicates reduced divergence from baseline power in late trials, significant in theta-band at 0…100 ms. Correspondence of the significant frequencies in FFT and ERSP analyses suggests that relative trial-wise power increases (in ERSP) are reduced due to a higher overall level of power. Panel (C) The ERP image of early and late subsets, showing the relatively greater positivities in early trials. No statistical testing was done as this image is for exploratory purposes. Panel D: The ITC of early and late subsets shows relative reduction in phase-locking in later trials, significant in the theta and low-alpha bands at ~200…300 ms.

Examining the FM theta-band power to test the effect of sustained attention, **H5fmt** is supported, theta-band power significantly higher in late than early trials by FFT analysis, *p* < 0.01; and significantly reduced trial-wise divergence of theta power by ERSP analysis, *p* < 0.05. The phase-locking analysis also shows significantly lower ITC for late than early trials in theta-band, *p* < 0.01. See Fig. 4 for visualisations.

### Primary Research Question

Finally, **HES** is strongly supported by larger ESs for congruency than for (no-)SCI questions, as shown below in Table 3.

**Table 3:** The numerical and nominal comparison of effect sizes for significant results pertaining to questions of (no-)SCI compared to questions of congruency.

Result comparison	Median ES	Nominal ES
**H1rtv** v **H2err**	0.57 v 0.97	med. v large
**H4pN1, H4tN1, H4tP3a** v	1.05 v 2.12	large v huge
**H3P3a, H3P3b**		

## Discussion

Regarding the main question, relating interference inhibition to gestalt processing, I have shown (for the first time to my knowledge) that the congruency of intra-modal primers has stronger influence on processing than the task-defined target (comparing within-behavioural and within-neural results), in a classification task with complex stimuli such as gestalts. The result for relative ES of congruency and (no-)SCI questions shows quite strong support for **HES**.

The notion that intra-modal interference requires more neural processing than a clearly defined target has serious implications for the way attention is engaged in, e.g. online search or Technology-Enhanced Learning (TEL) software. For instance, websites are commonly constructed to capture attention to topics beyond the core content, with powerful effects on reading habits, leading some to posit a connection between ADHD and Internet Addiction^11^. The impact of distractors on attention, including for clinical studies of ADHD, has often been examined with multi-modal protocols, e.g. visual targets and auditory distractors^12^. Therefore, the **HES** result illustrates an alternative approach and PSICAT offers a novel open-source tool to probe such effects (pending complete validation by test-retest and gold-standard comparisons).

The result for **HES** was based on comparing several tests which elucidated the functioning of PSICAT. Below I discuss behavioural and neural results of these tests in turn; but first I describe the relevant state of the art on gestalt image studies.

### Testing Natural Attention

Many existing CPTs assume the principle that sustained attention is an isolable faculty, independent of bottom-up distractor effects, and separate from potential deficits of stimulus processing in the tested modality. However rather than a single function, sustained attention is a set of cognitive processes which aim to maintain task-focused awareness, specifically: to keep the autonomic nervous system sufficiently aroused, and to orient attention toward the task at hand^3^. Clayton^4^ suggested that three kinds of cognitive processes contribute to this:

1. monitor and evaluate ongoing cognitive processes,
2. maintain task-relevant processes through resource allocation,
3. inhibit task-irrelevant processes.

In natural settings any or all of these processes may be impaired. Ecological stimuli tend to have compound features, requiring a composition of processes for attending. In visual feature space, natural stimuli are composed of global and local elements requiring integration^13^. Temporally, natural stimuli are related by causal structure, which creates priming effects and provides the opportunity for statistical learning. Hierarchically, natural stimuli are related by categorical membership.

For example, all these relationships are on ecological display in a group of prey animals (e.g. see https://www.flickr.com/photos/jurvetson/5905666047, not reproduced for copyright reasons). Such a scene consists of a global percept, the herd; formed of local elements, several members of the same species (e.g. *Equus quagga*). The herd interactions (feeding, fighting, etc), and hierarchical inter-relations (infant-parent or smaller-larger prey), all change over time. Task-relevant target stimuli (choosing mate/prey) are also commonly accompanied by various forms of intra-modal noise, e.g. distortion, occlusion; as well as multi-modal distractors. As stated by Wagemans, “*camouflage and camouflage breaking provide an ecological rationale for [gestalt] principles*”^14^.

Gestalts have been well-studied in the literature, dating back over 100 years with a recent resurgence of interest. In a comprehensive review, Wagemans and colleagues^14,15^ describe the conceptual and theoretical foundations of the gestalt approach to perceptual science, including implications for attention.

Gestalt imagery and Kanizsa SCIs have been of particular interest in vision research (for review see^16^). Ringach and Shapley^17^ showed that recognition of the gestalt percept involves the complete visual pathway in the brain. As a whole, the neural process of SCI perception has been described as reciprocal, where later visual areas segment the surfaces and assign boundaries needed to perceive illusory objects, and feed the surface/boundary signals back to early visual areas. These signals are then interpreted as an SCI, sometime after the inducer signals are perceived. Kogo *et al*.^18^ provide a model to describe this complete SCI formation process. This model implies that SCIs cannot be perceived without awareness of the inducing context, i.e. the Pac-Man shapes. A recent masking-controlled study of the perception of Kanizsa stimuli showed strong evidence to support that conclusion^16^. The ‘awareness dependence’ shown in these works implies that inattention would prevent SCI perception.

These findings together imply that: (a) SCIs are useful stimuli for testing attention with a more complete evocation of realistic neural processing; and (b) each SCI stimulus (PSICAT trial) will comprehensively test the visual attention mode, as opposed to only early areas for recognition of simple shapes. Based on this, I contend that over the whole duration the test will validly tap sustained attention.

Gestalt imagery is used in a number of clinical neuropsychological tests, but the emphasis is largely on testing peceptual function. (An overview of such tests is maintained at http://www.gestaltrevision.be/en/master-index/83-resources/reference-guides/). A notable test of this kind is L-POST^19^ which is computerised, freely available, and allows access to raw data.

Thus, although many papers have studied perceptual properties of gestalt imagery (showing that Kanizsa SCIs require awareness^16^ and are thus good for attention testing), few publications have used Kanizsa SCIs to study attention. In one example, Sokhadze and others^20^ used Kanizsa SCIs to test the ERPs of ADHD children, finding delayed early components in response to target stimuli and also that the delayed P3a component was better represented in the right hemisphere. However, code has not been released for any published protocol, to my knowledge.

### Neural Correlates of Gestalt Imagery

Kanizsa images have the added benefit that, despite being a complex percept, they are composed of simple geometric forms which allows manipulation and control of lower visual features, facilitating vision research modalities such as eye-tracking or brain imaging.

For instance, Tanskanen *et al*.^5^ demonstrated that stimuli with global contours are processed differently to those with equivalent local visual features but no contours, in later visual areas during 130…300 ms. The most pronounced and earliest difference was for a stimulus with a gestalt property where global contours are integrated to a single whole, which consequently showed the fastest reaction times.

In agreement with^5^, a study by Harris and others^21^ demonstrated that the Kanizsa SCI percept is generated at a later stage of visual processing. This was shown by participants’ inability to discriminate orientation of a Kanizsa triangle SCI when the inducers (Pac-Men) were masked from attention; whereas using the same method to mask the context of a simultaneous brightness contrast illusion did not affect the illusory percept. More evidence came from Murray *et al*.^22^, who proposed that SCI sensitivity in early visual areas V2 and V1 is mainly due to feedback modulation from higher areas. Indeed, Kanizsa SCIs have been shown to reliably activate the higher extrastriate cortex even when manipulation of visual features, such as rounding corners of the Pac-Man inducers, reduced the SCI percept strength^17^.

On the other hand, the SCI perception is not merely top-down classification, as illusory contours have been shown to be processed by binocular neurons in early visual cortex^23^.

As described above, SCI perception is a reciprocal process: later visual areas enable perception of illusory boundary signals, then feed these signals back to early visual areas, leading to interpretation as an SCI. Thus, SCIs cannot be perceived without awareness of the inducing context^16,18^.

Additionally, the neural signature should tell whether the SCI was ‘invisible’ due to inattention. Schurger and colleagues^24^ have shown that repeated stimulus presentation creates reduced variability in post-stimulus cortical activation, predictive of whether the stimulus was subjectively seen, suggesting that “conscious perception may involve the transient stabilization of distributed cortical networks, corresponding to a global brain-scale decision.” This suggests that the neural signature of trials should differ if the participant actually perceives the SCI or not, potentially providing a more accurate measure than behavioural responses (given that chance level of responding is 50%). Isolating the relevant global neural signal for analysis is another matter (which is why this question has been left as future work).

Another implication of the Kogo^18^ model affects the PSICAT primers: because SCI formation is relatively slow, the primer has small chance to induce SCI. Ringach’s results^17^ on reduction of SCI perception strength due to alteration of inducer visual properties also supports the notion that the primers, being not complete Pac-Man inducers, will not easily induce SCI. Thus, primers should play the role to prime participants to expect particular classes of Kanizsa shapes (SCI or no-SCI) without forming a ‘false-positive’ SCI. Schwarzkopf and Rees^25^ previously illustrated that response times were faster when gestalt targets were preceded with shape-congruent primes. Wasserstein^26^ investigated four perceptual closure tests, including SCIs, in a sample of focal lesion patients; they found that SCI processing was right-hemisphere dependent.

### Discussion of Behavioural Results

Natural visual attention, e.g. scanning a herd of *Equus quagga* (see Appendix A of How and Zanker^27^), requires processing of hierarchies both at input, (compositing scenes from low-level features), and at representation (attention can be directed to the global or local level). The integrated functioning of global and local processing is important for object recognition and contextualisation, [^28^ p.75]. One aspect of integration is feature saliency, which refers to an order of importance attached to features of the scene by the visual attention system, making certain features ‘stand out’. Order of processing (for sudden-onset stimuli) is ~25–50 ms. Task-related or top-down visual attention can modulate attention but the order is much slower, ~200 ms or more^29^.

The Kanizsa imagery allows both classes of primer and target stimuli, SCI or no-SCI, to have nearly equal spatial and luminance frequency characteristics. To distinguish between them thus depends more on the implicit shape than on the visual characteristics of the target itself, implying that the visual salience feature of implicit shape^30^ guides attention.

Thus, the results for response time and error-rate generally confirm the expectations regarding stimulus and congruency condition effects. Namely, congruent trial responses are faster and less error-prone than incongruent trials; while SCI targets are faster and more consistent than no-SCI targets. Relating back to the main question, Fig. 1 shows that the difference of means of congruency conditions is greater than the difference of means of gestalt conditions (because all congruent condition means are smaller than all incongruent condition means).

### Discussion of Neural Results

Various methods have been used to examine the neural correlates of sustained attention from electrophysiology data. In the time domain these include, e.g. ERPs^31^ and phase dynamics^32^; and in frequency domain there are, e.g. ERSPs and frequency-band power^33^.

#### Neural correlates of trial-wise attention

The main method used to examine neural correlates of the PSICAT trials were ERPs. I primarily followed recent work that showed enhanced attention capture by gestalt stimuli, with greater amplitude for early components N1, N2pc and P3a (though no difference for P1) compared to matched control stimuli^6^.

The visual N1 is the first negative peak after P1, widely distributed over the entire scalp but peaking at frontal regions before posterior. Based on this and on studies such as^9^, it is suggested that visual N1 is related to a discrimination process within the focal area of attention.

The visual N2pc is a very well studied component related to selective attention, known to appear posterior-contralateral to the focus of attention. In this case the PSICAT protocol presents stimuli centrally and N2pc is not elicited systematically.

The P3 is among the most widely studied components, a positive wave appearing between 250 to 500 ms^33^. The P3 is an endogenous component independent of the physical properties of the eliciting stimulus, implying it is related to decision making and attention. P3 comprises two sub-components, P3a and P3b, which index separate cognitive processes related to attention^10^. The P3a shows largest amplitude over frontal/central electrode sites from 250–280 ms; whereas the P3b is maximal in parietal areas from 300–500 ms. P3a and P3b also show distinct patterns of amplitude, latency and neuropharmacology^10^. John Polich proposes an explanatory model where P3 represents an early attention process: first an orienting change to a frontal working memory representation produces the P3a; this attention-driven stimulus signal then passes to temporal-parietal structures to create the P3b^10^.

Against this background, the ERP results strongly support **HES**, with very large ES from the contrasts in P3a,b (see Fig. 2). The scalp map for congruent P3a also indicates that this condition has a strong left-hemisphere bias, unlike other conditions. This seems to contradict earlier work^20,26^, which however studied clinical samples. In healthy individuals, it is possible that SCI classification becomes a categorical task when defined in a bounded context such as this experimental protocol, and thus processed in left cortex^34^; rather than the right-hemisphere localised gestalt integration task one would expect. The scalp map for incongruent P3a conversely shows strong occipital negativation, indicating the processing of novel visual stimuli.

**H4pN1** (and^6^) state that N1 in response to primers should follow the inequality *pri*.*SCI*.*N*1 < *pri*.*noSCI*.*N*1. Results support this with large ES. However, the early components after target onset are less clearly enhanced by SCIs. This seems to be due to the primers, as follows. Although incongruent SCI has more negative N1 than incongruent no-SCI, there is a close morphological match of incongruent SCI to *congruent* no-SCI from −100…200 ms (see Fig. 3, panel A, right plot), and these latter conditions share a no-SCI primer. This suggests that for these conditions, the no-SCI primer is the main influence on neural processing until after the target N1. Such a view accords with the thesis of^6^ and the result for **H4pN1**, that SCI primer shapes capture attention and are processed earlier, and consequently are overridden by following targets at an earlier time compared to no-SCI primers. The P3a results for **H4tP3** are not significant (perhaps due to the window of comparison, which was not chosen to maximise difference) but visually show clear differentiation, see Fig. 3, panel B. The results are consistent across congruency conditions, suggesting that the effect of primers is no longer dominant at this latency. This view is supported by examining the difference curves, (Fig. 3, panel A bottom), where the black curve for congruent SCI, no-SCI difference is closely mirrored until 300 ms by the inverse incongruent curve; i.e. *the incongruent primer SCI, no-SCI difference*.

#### Neural correlates of sustained attention

Sustained attention involves cooperation of a number of anatomical areas. For example, upon detection of inadequate attentional focus the posterior medial frontal cortex (pMFC) sends a signal via lateral prefrontal cortex (LPFC) to lower level sensory areas^4^. pMFC includes the anterior cingulate cortex (ACC) and dorsomedial prefrontal cortex (dmPFC).

Such anatomical inter-areal communication during sustained attention indicates the role of cortical oscillations. An explanatory model is given by Clayton^4^, where fronto-medial theta (4–8 Hz) power is suggested to reflect cognitive control processes, which monitor errors due to attentional lapses. These control processes are seen as fronto-posterior power-phase coupling to promote localised gamma oscillations (30–100 Hz) in task-relevant areas, and alpha (8–12 Hz) oscillations in task-irrelevant areas.

However others have suggested that fronto-medial theta reflects conditions of high working memory load^35^; or mental fatigue^36^. Indeed, fronto-medial theta power tends to increase across the duration of sustained attention tasks. This increase correlates with more commission errors and slowing of post-error RTs^36^: all suggestive of fatigue or excessive load^37^.

Thus fronto-medial theta is both associated with cognitive control and also increased by fatigue and error. This apparent contradiction is addressed by Clayton^4^, who suggests that fronto-medial theta responds to detection of inattention errors by attempting to compensate with increased signalling; to which however fatigue-depleted networks may be unable to respond.

Clayton^4^ follows Klimesch^35^ in suggesting that increased oscillatory alpha power in specific sensory areas is indicative of inhibition of task-irrelevant areas. However another view was proposed by Palva and Palva^32^, in which the observed alpha increase plays a direct role in sustaining mental representations, through mechanisms of power-phase coupling within the fronto-parietal network (FPN). Under this view, supposedly task-irrelevant areas are in fact supportive of the task, which reduces the need to categorise large areas of cortex as ‘task-irrelevant’ and in fact enriches the framework from Clayton^4^.

The results for sustained attention analysis are as expected: theta power is elevated in the last 10% of trials, compared to the first 10% (Fig. 4, panel A). ERSP indicates that this elevation most strongly affects first ~60 ms post-target onset (Fig. 4, panel B), dampening the event-locked power increase over baseline. The strong phase-locking at 5–7 Hz in early trials (Fig. 4, panel D, left plot showing ~0.27 ITC), is quite diminished by late trials, significantly (*p* < 0.01) after ~200 ms. This implies diminishment of attending^38^ in late trials, which in turn suggests that FM theta-band power does not increase due to, e.g. working memory load^35^, but due to genuine mental fatigue^37^. Indeed, because PSICAT is a context with little to no working memory load, it is more logical that increased FM theta is due to mental fatigue from sustained attention. The ERP image (Fig. 4, panel C) also indicates that event-locked amplitude diminishes from early to late trials, quite consistently across all trials in each subset.

In summary, prior work has pointed out a ‘lacuna’ in our understanding of integrated higher cognition^39^. This report (along with the PSICAT protocol) is a step toward improving that situation; but further studies, with a broader range of brain imaging techniques or clinical populations, are still required.

## Conclusion

The aim of this work is to move toward deeper insights into higher cognition and its neural correlates. Gestalt imagery, including Kanizsa shapes, is a class of visual stimuli that enables perceptual inference of implied images. Thus gestalt perception logically involves a more extensive integrative network than would perception of similar visual features with no gestalt property (as has also been shown empirically, see the section on Gestalt imagery above). For this reason I designed the PSICAT protocol based on the presentation of Kanizsa stimuli targets, built from Pac-Man–like shapes that induce an SCI if angled collinearly, and preceded by acute-angle line primers.

PSICAT’s primer-target stimuli pairs are either congruent and saliency reinforcing, or incongruent and task-interfering. Primer congruency thus modulates the saliency of edges in the SCI. Being task-irrelevant, primers constitute a case of target-interference. When the interference is presented congruently, the primer contours evoke the same processing response as the following target stimulus. Thus, saliency is reinforced. When incongruent, primers clearly reinforce the opposite class to the target stimulus and induce a requirement for interference inhibition. Behavioural and neural results show clearly and in detail that the congruency effect on processing is considerably greater than the target-selection effect. This result has implications for the design of technology and user interfaces.

While extending the state of the art for CPTs of sustained attention, the PSICAT protocol aims to remain as simple as possible to prevent unwanted confounds; but it is open to customization. Open-source code hosted on a public repository (https://github.com/zenBen/Kanizsa_Prime/) makes it possible to freely use or adapt the PSICAT functionality, including the capacity to generate geometrically defined SCI shapes in Neurobs Presentation format. This can serve as the basis for entirely new protocol designs. The code is under MIT’s open-source licence allowing others to freely use the PSICAT protocol as described, or repurpose any part thereof as they see fit.

In summary, this paper makes two main contributions:

1. Novel results to **HES** show that task-irrelevant incongruency of an intra-modal distractor prime can evoke stronger behavioural and neural responses than the task-relevant stimulus condition.
2. The open-source PSICAT protocol provides a well-controlled, novel test of sustained attention.

## Methods

### PSICAT

The PSICAT protocol has the following novel main features:

1. SCI target trials are contrasted with targets composed of similar Pac-Man inducers, rotated to disrupt collinearity and prevent inducing a polygon (no-SCI).
2. Targets are preceded by a *primer stimulus* (see Fig. 5), which is either congruent (same inducer angles) or incongruent (different inducer angles) to the target stimulus. Collinearly angled primers do not induce an SCI as strongly as the Pac-Man inducers, but do reinforce the salient features of the following SCI - i.e. the inducer angles. Priming of targets thus manipulates the participant’s saliency processing.

**Figure 5:**
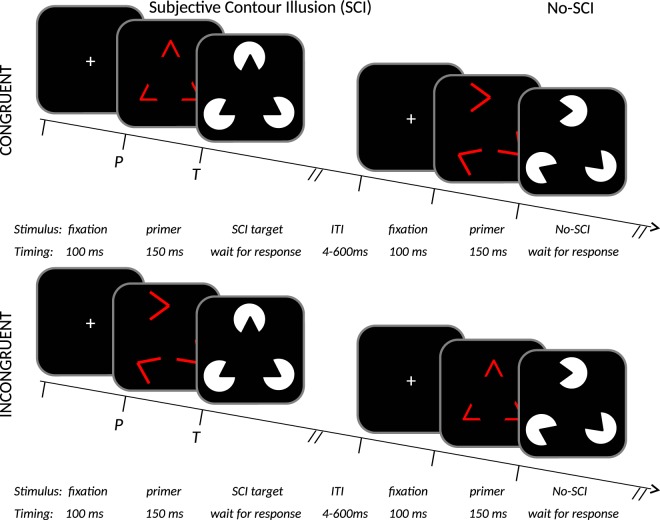
Schematic of the stimuli and protocol structure used in PSICAT. Stimuli are exemplified top left by a triangular Kanizsa subjective-contour-illusion target, displayed white-on-black; with the respective congruent red-line primer. Single trials of the protocol are shown in 2 × 2 conditions: congruent/incongruent × SCI/no-SCI. Congruent primer angles match the following target’s angles, incongruent do not match. Trials from each condition are distributed uniformly at random. Participant’s task is to classify whether targets are SCI or no-SCI; the primers are to be ignored. PSICAT consists of 550 trials in five blocks, and requires ~22 minutes to complete.
3. The protocol randomly generates irregularly-shaped SCIs/no-SCIs (i.e. vertices have arbitrary, though constrained, angles), which prevents participants from learning to classify by minor salient features. For example, in trials with a regular SCI one particular spot on the screen would be occupied by a Pac-Man vertex at a fixed angle; this could be used to discriminate from no-SCIs by focusing on the angle.
4. The protocol presents both 3- and 4-vertex stimuli, manipulating the quantity of visual information onscreen. This allows separation of the effect of illusory shape (triangle, quadrilateral) from target class (SCI, no-SCI), and also contributes to point 3 above. Vertex number adds a further dimension (for control purposes), giving eight conditions in total; however because there is no task-relevance to the vertex number it is not a research question and I reasonably exclude vertex number from current analyses.
5. The open-source code defines stimuli as geometric objects and thus allows users the complete control of parameters: visual angle, timing, luminosity, etc.

#### PSICAT Presentation code

In order to create these stimuli with the features described above: irregular shapes, congruent primers, variable vertices; and present them with required randomisation, a comprehensive ‘Kanizsa-making’ code-base was built for the Neurobs Presentation platform for psychophysics experiments https://www.neurobs.com. The code and documentation is available from github at https://github.com/zenBen/Kanizsa_Prime/.

This repository allows users to implement the PSICAT protocol exactly as described, but further allows alteration of parameters (timing, conditions), and creation of custom Kanizsa shapes and primers via geometric parameters. The latter enables researchers to develop any form of Presentation experiment with such stimuli.

Briefly, the code consists of two ‘.sce’ files (the Presentation format describing scenarios for experiments), for practice and test versions. There are also five Program Control Language ‘.pcl’ files. PSICAT.pcl defines the particulars of the protocol, such as number of blocks and trials, timing constants, color, etc. This is the code of interest for researchers who want to use the protocol as is, changing only protocol-specific parameters. Three of the other.pcl files contain the functions to generate Kanizsa stimuli. This is the code of interest to users who need such stimuli, whether for use in the PSICAT protocol or another of their own design.

Also in the repository are several images and audio files for the participant’s instructions (in Finnish by default but can be localised by the fifth.pcl file). An example experiment file, ‘PSICAT.exp’, demonstrates how to set up the protocol in Presentation.

#### PSICAT Protocol

PSICAT is a two-alternative forced-choice task, with lateral responses to the presence or absence of an SCI target. Handedness of response is required to be counter-balanced, i.e. randomised across participants.

The protocol begins with an explanation and set of 12 practice trials, three for each condition, to brief participants on the task under supervision of an experimenter. PSICAT consists of five blocks of 110 trials, with a one minute rest break between each block. In each block, trials were uniformly randomly distributed between 2 × 2 × 2 conditions: congruent vs incongruent × SCI vs no-SCI × 3- vs 4-vertex.

Example trials are shown in Fig. 5. Each trial consists of a preparatory fixation cross, primer-target pair, and inter-trial interval (ITI). Fixation lasts 100 ms; primers are flashed for 150 ms; targets are held until the participant responds (~750 ms on average in this study); and ITI is 500 ± 100 ms, (varied to minimise presentation expectancy which reduces trial effects). Thus an average trial should last ~1.5 sec, giving an estimated protocol duration of ~22 mins, similar to T.O.V.A.

### Validation experiment

The operation of PSICAT has been validated by deployment in a clinical trial^40^, where it was used to test adult ADHD participants alongside the healthy participants who are reported herein (the clinical results are to be reported elsewhere; this context is mentioned merely for full disclosure).

#### Participants

17 neurologically healthy participants were recruited by word of mouth, and were remunerated. They had normal or corrected-to-normal vision, were all right-handed, and were not taking any medication. There were 11 females, 6 males; average age was 34 (standard deviation 11). Participants provided written informed consent before entering the study. The protocol followed the Declaration of Helsinki, and ethical approval was obtained from The Ethical Committee of the Hospital District of Helsinki and Uusimaa, 28/03/2012, 621/1999, 24 §.

#### EEG recording

In an electrically shielded room, EEG data was continuously recorded (DC–104 Hz; sampling rate (SR) 512 Hz) using Biosemi ActiveTwo amplifier, with 128 active electrodes mounted in a fitted flexible cloth cap according to the Biosemi 128 montage. Offline reference electrodes were attached at the mastoids. Electrooculography was measured with two bipolar electrode sets, mounted horizontally at the outer canthi of each eye, and vertically above and below the left eye.

Additionally, electrocardiography was measured from chest-mounted electrodes at the manubrium and lower left rib. Electrodermal activity was measured from the proximal phallanges of the index and forefinger on the non-dominant hand. These autonomic nervous system signals are not analysed herein.

Biosemi ActiveTwo equipment reduces the need for impedance measurement (http://www.biosemi.com/faq/cms&drl.htm). Instead, the quality of contact between electrode and skin was monitored using running average voltage offset at each electrode, which was kept below ±25 mV.

#### Procedure

Participants were tested with PSICAT as the second in a battery of tests. The whole session consisted of: briefing, dressing in the electrodes, passive baseline measurement (4 minutes), T.O.V.A. CPT (22 minutes), PSICAT protocol (~22 minutes), eyes-closed vigilance protocol (~15 minutes), and post-test baseline (4 minutes). The recording period lasted just over one hour.

### Analysis

#### Data preprocessing

Behavioural data was obtained from the Presentation log file, and analysed in the R platform for statistical computing^41^.

EEG data was imported to Matlab (Natick, MA) using the EEGLAB toolbox^42^. The data was preprocessed using batch scripts powered by CTAP^43,44^, in the following steps:

- Highpass filtering at 0.5 Hz, using Matlab’s least-squares linear-phase finite impulse response (FIR) filter design, with filter order of 1.5 × SR, frequency characteristics [0 $\frac{0.85}{SR}$ $\frac{1}{SR}$ 1], and amplitude characteristics [0 0 1 1].
- Bad channel detection by the union of: visual identification made during the recording session, and automated classification using the FASTER toolbox^45^. Detected channels were removed (per participant *M* = 3.6, *SD* = 2.2).
- Bad epoch detection by automated classification with the FASTER toolbox^45^. Detected epochs were removed (per participant *M* = 19, *SD* = 5).
- Independent Components (IC) Analysis using the extended Infomax algorithm after^46^.
- Bad IC detection using the union of FASTER^45^ and ADJUST^47^ automated classifcation. Detected ICs were removed and their activations subtracted from the EEG data using the EEGLAB function pop_subcomp().
- Spherical interpolation of rejected channels.

#### EEG analysis

ERPs were generated from the EEG data using Matlab, time-locked to the target onset of hit trials only (excluding error trials). Continuous EEG was split into 750 ms epochs, with 100 ms pre-stimulus baseline, primer lasting 150 ms, and 500 ms after target stimulus onset. Epochs were baseline-corrected with respect to the mean voltage of the 100 ms period *preceding the part of the epoch relevant to the research question*. Specifically, for both **H3**s and for **H4pN1**, the baseline was −250…−150 ms – the 100 ms preceding the whole trial. While for **H4tN1**, **H4tP3** the baseline was −100…0 ms – i.e. the latter 2/3 s of the primer presentation. Although this is more complex than using a single baseline period for all ERPs, it was necessary to control (on a trial-by-trial basis) for the variability induced by primer presentations in the ERPs for target presentations. After baseline-correction, a 20 Hz low-pass filter was applied for visualisation and testing.

To address **H3P3a,b**, ERP curves P3a and P3b were generated for ROIs chosen based on^10^: for P3a, the vertex plus five closest electrodes (A2 B1 C1 D1 D15); and for P3b, midline parietal electrode A19 (equivalent to Pz under 10/20 system) plus four closest electrodes (A4 A5 A20 A32). See Fig. 2 for ROI locations. These ERPs were averaged in congruent and incongruent conditions separately.

To address **H4pN1,tN1,tP3**, curves for early components were generated at a region of interest (ROI) centered on A23 (equivalent to Oz under 10/20 system) plus A15 A22 A24 A28 (i.e. four closest electrodes). This ROI was chosen to broadly reflect all early visual-area activity.

To address **H5fmt**, epoched EEG data was subsetted to the first 10% and last 10% of epochs (10% was chosen to emulate the approach in^7^). Using EEGLAB’s graphical user interface, spectral power was estimated within each of these *early* and *late* subsets for all trials (i.e. pooling all conditions), and compared by two separate methods. First, the Fast Fourier Transform (FFT) estimate of spectral power was computed (using EEGLAB’s pwelch() method) for the region of interest C19-C21 (C20 = Fz in the 10/20 system, plus adjacent midline electrodes). Second, ERSP was computed at C20 alone (using the timef() method implemented in EEGLAB). ERSPs measure signal power relative to a baseline, expressed in decibels, and so were plotted and tested against the average baseline of combined early and late subsets, to control for signal drift from beginning to end of the CPT. Due to the constraints of the epoch size and the desired frequency range (i.e. lower frequencies cannot be calculated without having more ‘pad’ data around the epoch of interest), ERSP duration was limited to 0…300 ms. The FFT shows the absolute difference in spectral power at every frequency, thus supporting interpretation of the ERSP, which only shows relative power but reveals the temporal dynamics. Lastly, the inter-trial coherence (ITC) measure of stimulus-onset phase locking was estimated at C20 for the same time range as ERSP, to assess phase coherence across trials^48^. ITC has been positively associated with relative levels of attending to a trial-wise targets^38^, and thus should help clarify the effect of FM theta power changes.

#### Statistical methods

For testing **H1rt** and **H1rtv** I first removed error trials; participant-wise median errors per condition was *con*.*SCI* 1; *con*.*noSCI* 1; *inc*.*SCI* 3; *inc*.*noSCI* 1. For **H1rt** I analysed participant-wise median RTs in order to control outliers and obtain a robust central tendency estimate^49^. Although still a biased estimator, condition-bias will be approximately equal when the number of trials per condition is equal, which is true here due to the low error rates. For **H1rtv** I analysed participant-wise variability of RTs.

The paired samples for each comparison were tested for homogeneity of variance by Levene’s test, passing in all cases. Samples were all shown to be strongly non-normal by Shapiro-Wilk test, indicating a non-parametric test. Condition differences were thus tested by pairwise one-sided Mann-Whitney U-tests. ESs for these tests was calculated by $ES=z\frac{1}{\sqrt{N}}$, where *z* is the test z-score and *N* is total number of observations (after, [^50^ pp.550]).

ERP amplitude was estimated as the mean across a window, instead of selecting a peak value, as recommended by Luck^51^. Window length was fixed proportional to the component duration, i.e. 20 ms for N1 and 40 ms for P3a,b. Window latency was centered on the mean peak latency of the relevant grand average ERPs. Thus condition- and participant-wise mean amplitude was calculated within windows: primer N1 0 ± 10 ms; target N1 165 ± 10 ms; P3a 280 ± 20 ms; P3b 480 ± 20 ms. The resulting values were tested for condition-wise difference using paired-sample one-tailed *t*-test. Cohen’s *d* ES was computed for these results using the ‘compute-es’ package for R platform^52^.

To estimate the statistical difference of FM theta-band power in first and last 10% of trials, I used EEGLAB’s permutation testing procedure. This constructs a distribution for the null-hypothesis of no difference in conditions, and conducts an (unpaired) test for whether the observed value lies in the tails of the distribution. This procedure was applied (using EEGLAB) for power spectrum, ERSP, and ITC comparison. Resulting significant frequencies × times are indicated in plots accompanying those data visualisations. Permutation testing gives stochastic output for exact regions of significance, so calculations were repeated ten times to ensure that what is displayed is characteristic of a repeatable pattern.

Finally, to test whether ESs from above results were larger for congruency than for (no-)SCI questions, I compared the pooled absolute values of ESs from significant results, separately for behavioural and neural data sources (avoiding mixing ES-estimation methods). This gave the following *(no-)SCI vs congruency* comparisons: **H1rtv** vs **H2err**; [**H4pN1**, **H4tN1**, **H4tP3a**] vs [**H3P3a**, **H3P3b**]. To compare, I took the median ES value and substituted the nominal ‘rule-of-thumb’ value (small, medium, large, extended according to^53^ for Cohen’s *d*) to demonstrate the inequality. Because ES values are standardised, there was no need to do further statistical testing.

For each family of analyses, family-wise error rate was controlled by *p*-value adjustment using the Holm-Bonferroni method.

## Data Availability

The anonymized behavioural and physiological dataset analyzed herein is available via a permanent object identifier hosted by the scientific data repository Figshare at https://figshare.com/account/projects/28047/articles/5759487.
